# Impact of COVID-19 on Acute Stroke Presentation in a Designated COVID-19 Hospital

**DOI:** 10.3389/fneur.2021.673703

**Published:** 2021-09-08

**Authors:** Qing Tan, Qing-Jun Liu, Wen-Hui Fan, Xiao-Yan Du, Lin Wu, Hong-Min Gong, Jing Wei, Rui Zhao, Ming Lei, Li-Bo Zhao

**Affiliations:** ^1^Department of Neurology, Yongchuan Hospital, Chongqing Medical University, Chongqing, China; ^2^Chongqing Key Laboratory of Cerebrovascular Disease Research, Chongqing, China; ^3^Department of Neurology, Chongqing Ninth People's Hospital, Chongqing, China

**Keywords:** COVID-19, coronavirus, acute stroke, designated hospital, stroke center

## Abstract

**Objectives:** Thousands of designated COVID-19 hospitals have been set up in China to fight the ongoing COVID-19 pandemic. Anecdotal reports indicate a falling rate of acute stroke diagnoses in these hospitals during the COVID-19 period. We conducted an exploratory single-center analysis to estimate the change in acute stroke presentation at the designated COVID-19 hospitals.

**Methods:** This retrospective observational study included all patients admitted to Yongchuan Hospital Affiliated to Chongqing Medical University with acute stroke between January 24 and March 10, 2020. Patient demographics, characteristics of the stroke, treatment details, and clinical outcomes were compared with those of patients admitted in the corresponding period in the year before (2019, “the pre-COVID-19 period”). Subgroup analysis was performed in the ischemic and hemorrhagic stroke groups.

**Results:** A total of 110 patients presented with acute stroke symptoms during the COVID-19 pandemic, compared with 173 patients in the pre-COVID-19 period. A higher proportion of stroke patients presented to the hospital *via* emergency medical services during the pandemic (48.2 vs. 31.8%, *p* = 0.006). There was a lower proportion of ischemic stroke patients (50.9 vs. 65.3%, *p* = 0.016) than in the preceding year. There were significantly fewer patients with 90-day modified Rankin Scale score ≥3 in the COVID-19 period compared with the pre-COVID-19 period (17.3 vs. 30.6%, *p* = 0.012). Among patients with ischemic stroke, the mean time from patient arrival to vessel puncture for emergency endovascular therapy in the COVID-19 period was shorter than that in the pre-COVID-19 period (109.18 ± 71.39 vs. 270.50 ± 161.51 min, *p* = 0.002). Among patients with hemorrhagic stroke, the rate of emergency surgical operation in the COVID-19 period was higher than that in the pre-COVID-19 period (48.1 vs. 30.0%, *p* = 0.047). The mean time from patient arrival to emergency surgical operation (15.31 ± 22.89 vs. 51.72 ± 40.47 min, *p* = 0.002) was shorter in the COVID-19 period than in the pre-COVID-19 period.

**Conclusions:** Although fewer acute stroke patients sought medical care in this designated COVID-19 hospital during the COVID-19 pandemic, this type of hospital was more efficient for timely treatment of acute stroke. Recognizing how acute strokes presented in designated COVID-19 hospitals will contribute to appropriate adjustments in strategy for dealing with acute stroke during COVID-19 and future pandemics.

## Introduction

The COVID-19 pandemic, which originated in Wuhan, China, in December of 2019, spread rapidly worldwide and became a huge challenge for global health systems. By December 31, 2020, the global number of COVID-19 infections and related deaths had reached over 83 million and 1.8 million, respectively (1).

Acute stroke refers to a sudden cerebrovascular accident and is a medical emergency. Acute stroke can be divided into ischemic stroke and hemorrhagic stroke according to the occurrence of cerebrovascular blockage and cerebrovascular rupture, which is usually verified by imaging examination (2, 3). Thrombolytic therapy, endovascular therapy, and surgery within the time window have a significant impact on the prognosis of acute stroke patients (4–6). However, reports suggest that the number of acute stroke patients presenting to hospitals during COVID-19 was significantly reduced (7).

During the pandemic period, thousands of designated COVID-19 hospitals have been established in China to centralized and treat COVID-19 patients in a designated region. These hospitals are often designated by regional large tertiary care centers (8). While such designated hospitals have played an important role in fighting the pandemic (9), their stroke centers have been significantly affected. Yongchuan Hospital Affiliated to Chongqing Medical University (YCHCQMU) is both a comprehensive stroke center and one of the four designated hospitals for centralized treatment of COVID-19 patients in Chongqing, China. As of March 1, 2020, a total of 90 confirmed COVID-19 patients were admitted to this hospital (10). The purpose of this study was to evaluate our institution's experience as a designated COVID-19 hospital with acute stroke patients during the early months of the COVID-19 pandemic.

## Methods

### Study Design

Acute stroke patients who presented to YCHCQMU were retrospectively identified and compared. YCHCQMU is a 1,480-bed, tertiary care, comprehensive stroke center in western Chongqing. Stroke includes ischemic stroke (cerebral infarction) and hemorrhagic stroke (parenchymal hemorrhage, intraventricular hemorrhage, and subarachnoid hemorrhage). All the onset of acute stroke was within 2 weeks. Acute stroke was clinically diagnosed by a vascular neurologist and verified by radiographic evidence: computed tomography (CT) or magnetic resonance imaging. Patients were divided into two cohorts according to treatment periods: (1) COVID-19, which included patients admitted on dates between January 24 and March 10, 2020. This period was chosen to coincide with the experience of Chongqing initiating a first-level response to major public health emergencies. (2) Pre-COVID-19, which included patients admitted in the same calendar period, but in 2019. Subgroup analysis was performed in the ischemic and hemorrhagic stroke groups. The research was approved by the Medical Ethics Committee of YCHCQMU and was conducted in accordance with the 1964 Declaration of Helsinki and its later amendments or comparable ethical standards.

### Data Collection

The following clinical information was extracted from patients' medical records and follow-up records: (1) sex; (2) age; (3) home address; (4) time from last known normal (LKN) to hospital arrival; (5) arrival mode; (6) length of stay; (7) final diagnosis; (8) whether the patient had a refusal of medical care recommended by medical personnel (denial of medical services); (9) National Institutes of Health Stroke Scale (NIHSS) score on admission; (10) use and timing of thrombolytic agent, and/or emergency endovascular thrombectomy, and/or emergency surgical operation; (11) 90-day modified Rankin Scale (mRS); and (12) deaths at 90 days. In addition, 90 days mRS ≥3 points and <3 points indicate poor and good prognosis, respectively.

### Statistical Analysis

SPSS 22.0 statistical software was used for statistical analysis. Categorical variables were reported as proportions. Continuous variables were reported as medians with interquartile range, or means with standard deviation. Between-group comparisons for categorical data were made using chi-square or Fisher's exact test when contingency table cell counts were <5. The Kolmogorov–Smirnov test was used for the normal distribution test. Between-group comparisons for non-normally distributed continuous data were made using Mann–Whitney U-test or independent *t*-test for normally distributed continuous data. *p* < 0.05 was considered to indicate statistical significance.

## Results

### Comparison in the Acute Stroke Overall

A total of 110 patients presented with acute stroke symptoms during the COVID-19 period, in comparison to 173 patients in the pre-COVID-19 period (Table 1). All acute stroke patients were exclude with COVID-19 diagnosed by nucleic acid detection test. The mean age of patients was 68 ± 13 and 68 ± 14 years in the COVID-19 period and pre-COVID-19 period, respectively, with a low proportion of female patients (42.7 vs. 36.4%) and a low proportion of urban residents (30.0 vs. 34.1%). The mean time of LKN to hospital arrival was 243 (179–931) min in the COVID-19 period and 301 (179–1017) min in the pre-COVID-19 period. There was no statistically significant difference in any of these characteristics between the two periods. The proportion of patients who presented to the hospital *via* emergency medical services (EMS) during the COVID-19 period in comparison to the pre-COVID-19 period increased significantly (48.2 vs. 31.8%, *p* = 0.006). The proportion of patients diagnosed with ischemic stroke in the COVID-19 period was lower than that in the pre-COVID-19 period (50.9 vs. 65.3%, *p* = 0.016). Further, 20.9 and 22.5% patients rejected medical services in the COVID-19 period and the pre-COVID-19 period, respectively. There was no statistically significant difference (*p* = 0.746). The mean presenting NIHSS score was 12 ± 8 in the COVID-19 period and 13 ± 9 in the pre-COVID-19 period (*p* = 0.441). The number of patients with 90 days mRS ≥3 points was significantly lesser in the COVID-19 period than in the pre-COVID-19 period (17.3 vs. 30.6%, *p* = 0.012). There was no statistically significant difference in the rate of death at 90 days between the two periods (6.4 vs.9.8%, *p* = 0.308).

**Table 1:** Demographics, characteristics, and clinical outcomes of acute stroke patients in the COVID-19 period in comparison to the pre-COVID-19 period.

	**COVID-19**	**pre-COVID-19**	***t/Z/χ^2^***	***P* value**
	**(*n* = 110)**	**(*n* = 173)**		
Female, *n* (%)	47 (42.7)	63 (36.4)	1.127	0.288
Age (years), mean ± SD	68 ± 13	68 ± 14	0.096	0.924
Urban resident, *n* (%)	33 (30.0)	59 (34.1)	0.516	0.472
LKN to hospital arrival(min), IQR	243 (179,931)	301 (179,1017)	0.928	0.354
Arrival mode: EMS, *n* (%)	53 (48.2)	55 (31.8)	7.654	0.006
Length of stay (d), IQR	14.5 (8,20)	12 (7,17)	1.759	0.079
Ultimate diagnosis: Ischemic stroke, *n* (%)	56 (50.9)	113 (65.3)	5.804	0.016
Denial of medical services, *n* (%)	23 (20.9)	39 (22.5)	0.105	0.746
NIHSS at presentation, mean ± SD	12 ± 8	13 ± 9	0.771	0.441
mRS at 90 days ≥ 3, *n* (%)	19 (17.3)	53 (30.6)	6.330	0.012
Deaths at 90 days, *n* (%)	7 (6.4)	17 (9.8)	1.039	0.308

*COVID-19, coronavirus disease 2019; LKN, last known normal; EMS, emergency medical services; SD, standard deviation; IQR, interquartile range; Denial of medical services indicates the patient had a refusal of medical care recommended by medical personnel; NIHSS, National Institutes of Health Stroke Scale; mRS, modified Rankin Scale*.

### Comparison in the Acute Ischemic Stroke

There were no significant differences in these indicators between the two groups, including sex, age, home address, LKN to hospital arrival, arrival mode, length of stay, denial of medical services, NIHSS score on admission, the rates of thrombolysis and endovascular thrombectomy, the mean time from patient arrival to administration of thrombolytic drug (door to needle), mRS, and deaths at 90 days. However, the mean time from patient arrival to vessel puncture for emergency endovascular therapy (door to puncture) in the COVID-19 period was shorter than that in the pre-COVID-19 period (109.18 ± 71.39 vs. 270.50 ± 161.51 min, *p* = 0.002) (Table 2).

**Table 2:** Demographics, characteristics, treatment details, and clinical outcomes of acute ischemic stroke patients in the COVID-19 period in comparison to the pre-COVID-19 period.

	**COVID-19**	**pre-COVID-19**	***t/Z/χ^2^***	***P* value**
	**(*n* = 56)**	**(*n* = 113)**		
Female, *n* (%)	23 (41.1)	46 (40.7)	0.002	0.964
Age (years), mean ± SD	71 ± 13	68 ± 14	1.307	0.193
Urban resident, *n* (%)	21 (37.5)	45 (39.8)	0.085	0.771
LKN to hospital arrival(min), IQR	242.5 (181,599)	359 (185,1377)	1.518	0.129
Arrival mode: EMS, *n* (%)	21 (37.5)	27 (23.9)	3.409	0.065
Length of stay (d), IQR	10.5 (5.75,15)	11 (6,13)	0.884	0.377
Denial of medical services, *n* (%)	14 (25.0)	28 (24.8)	0.001	0.975
NIHSS at presentation, mean ± SD	9 ± 8	9 ± 8	0.148	0.882
Intravenous thrombolysis, *n* (%)	11 (19.6)	30 (26.5)	0.972	0.324
Door-to-needle (min), mean ± SD	52.45 ± 26.14	43.13 ± 34.27	0.822	0.416
Endovascular thrombectomy, *n* (%)	11 (19.6)	16 (14.2)	0.839	0.360
Door-to-puncture(min), mean ± SD	109.18 ± 71.39	270.50 ± 161.51	3.526	0.002
mRS at 90 days ≥ 3, *n* (%)	10 (17.9)	26 (23.0)	0.593	0.441
Deaths, *n* (%)	2 (3.6)	4 (3.5)	0.000	1.000

*COVID-19, coronavirus disease 2019; LKN, last known normal; EMS, emergency medical services; SD, standard deviation; IQR, interquartile range; Denial of medical services indicates the patient had a refusal of medical care recommended by medical personnel; NIHSS, National Institutes of Health Stroke Scale; mRS, modified Rankin Scale*.

### Comparison in the Acute Hemorrhagic Stroke

There were no significant differences in these indicators between the two groups, including sex, age, home address, LKN to hospital arrival, arrival mode, length of stay, denial of medical services, NIHSS score on admission, and deaths at 90 days. However, 48.1% of patients underwent emergency surgery in the COVID-19 period as against 30.0% patients in the pre-COVID-19 period. There was a statistically significant difference (*p* = 0.047). The mean time from patient arrival to emergency surgical operation (15.31 ± 22.89 vs. 51.72 ± 40.47 min, *p* = 0.002) was shorter in the COVID-19 period than in the pre-COVID-19 period. The number of patients with 90 days mRS ≥3 points was significantly lesser in the COVID-19 period than in the pre-COVID-19 period (16.7 vs. 45.0%, *p* = 0.001) (Table 3).

**Table 3:** Demographics, characteristics, treatment details, and clinical outcomes of acute hemorrhagic stroke patients in the COVID-19 period in comparison to the pre-COVID-19 period.

	**COVID-19**	**pre-COVID-19**	***t/Z/χ^2^***	***P* value**
	**(*n* = 54)**	**(*n* = 60)**		
Female, *n* (%)	24 (44.4)	17 (28.3)	3.203	0.073
Age (years), mean ± SD	65 ± 11	68 ± 13	1.116	0.267
Urban resident, *n* (%)	12 (22.2)	14 (23.3)	0.020	0.888
LKN to hospital arrival	243	119.75	1.235	0.217
(min), IQR	(178.25, 479.75)	(183.5, 554.75)		
Arrival mode: EMS, *n* (%)	32 (59.3)	28 (46.7)	1.808	0.179
Length of stay (d), IQR	18.5	20	0.650	0.516
	(12.75,27)	(13,29.25)		
Denial of medical services, *n* (%)	9 (16.7)	11 (18.3)	0.055	0.815
NIHSS at presentation, mean ± SD	20 ± 3	19 ± 4	0.623	0.535
Emergent surgical operation, *n* (%)	26 (48.1)	18 (30.0)	3.950	0.047
Door-to-surgery (min), mean ± SD	15.31 ± 22.89	51.72 ± 40.47	3.454	0.002
mRS at 90 days ≥ 3, *n* (%)	9 (16.7)	27 (45.0)	10.560	0.001
Deaths, *n* (%)	5 (9.3)	13 (21.7)	3.291	0.070

*COVID-19, coronavirus disease 2019; LKN, last known normal; EMS, emergency medical services; SD, standard deviation; IQR, interquartile range; Denial of medical services indicates the patient had a refusal of medical care recommended by medical personnel; NIHSS, National Institutes of Health Stroke Scale; mRS, modified Rankin Scale*.

## Discussion

The COVID-19 pandemic has had a huge impact on our hospital, especially in the early stages of the pandemic, when the hospital also served as a designated COVID-19 hospital. Studies in the international literature has generally reported a decline in hospital admissions during the COVID-19 period compared with the pre-COVID-19 period, due to the impact of COVID-19 pandemic on acute stroke care services. For example, Sacco et al. (11) found 24.36% decrease in Italy, Butt et al. (12) found 12.66% decrease in Denmark, and Kristoffersen et al. (13) found 29.91% decrease in Norway. Consistent with previous study, we found a 36.42% (63/173) decrease. In addition, a significant decrease was observed in the proportion of ischemic stroke between the two periods, which was consistent with the study of Tejada et al., which showed the weekly mean admitted ischemic stroke patients decreased during the pandemic period compared to the pre-pandemic period (124 vs. 173, *p* < 0.001) (14). In addition, the proportion of patients who presented to the hospital *via* EMS and the proportion of patients undergoing emergency surgery both increased significantly. All of these findings point to the fact that fewer acute stroke patients with mild symptoms sought medical care during the COVID-19 period. The decrease in patients with mild symptoms led to a decrease in the number of acute stroke patients, decrease in the proportion of ischemic stroke patients who tend to be less severe than hemorrhagic stroke patients (15), increase in the proportion of patients who presented to the hospital *via* EMS, and an increase in the proportion of patients undergoing emergency surgery. This is also consistent with the previous research results of Siegler et al. (16). Through a 6-month follow-up study, Siegler et al. found that the number of acute stroke patients who presented to the hospital in private cars during the COVID-19 period was 55% lower than that during the pre-COVID-19 period, and acute stroke patients who presented to the hospital in private cars tended to have less severe conditions. We identified two reasons for the decrease in acute stroke patients with mild symptoms: (1) worry about virus infection and transmission, as SARS-CoV-2 is highly contagious (17). Most acute stroke patients with mild symptoms expressed that their delay in seeing a doctor was related to the fear caused by COVID-19. The patients expressed that they would rather wait at home for the disease to improve or the pandemic to get under control (18). The other reason is the (2) increased screening measures: in order to prevent nosocomial cross-infection of COVID-19, screening measures increased during the COVID-19 period (19). The increased screening measures made it difficult for acute stroke patients to seek medical treatment.

Our study also showed that the proportion of acute stroke patients with poor prognosis during the COVID-19 period was significantly lower than that during the pre-COVID-19 period. In addition, the door-to-puncture time and door-to-surgery time, which refers to the in-hospital time, were significantly reduced compared to the corresponding period in the pre-COVID-19 year, which was consistent with previous findings. Nagamine et al. study (20) showed that the door-to-puncture time during the COVID-19 period was lesser than that in the non-pandemic period (109 ± 32.4 vs. 132 ± 22.6 min). Zini et al. (21) found that although the door-to-puncture time between the 2 years only had a slight reduction (116.9 ± 39 vs. 118.8 ± 55.7 min), the mean time from patient arrival to CT scan that applied to each patient in the hospital was increased. As a consequence, the time after CT scan to puncture that exclusively belonged to stroke centers was decreased. Lee et al. (22) found that the time from patient arrival to surgery in the COVID-19 period was higher than that in the pre-COVID-19 period, but in the comparison of the subgroup of emergency surgery, the door-to-surgery time during the COVID-19 period was significantly shorter than that during the pre-COVID-19 period (*p* = 0.04). However, our study showed that there was no significant difference in the numbers of emergency endovascular therapy between the two periods. In the context of the addition of rigorous COVID-19 screening programs, the abovementioned changes point to the fact that our stroke center was more efficient during COVID-19. As a result of the improvement of our stroke center work efficiency, on the one hand, patients could quickly complete a series of preoperative examinations, and the doctors could immediately arrange for the surgery, which led to the shortened door-to-puncture and door-to-surgery times. On the other hand, the treatment effect of acute stroke patients was greatly improved, which contributed to the significantly reduced proportion of acute stroke patients with poor prognosis. The reasons for the improvement of the stroke center work efficiency during the COVID-19 period may be as follows: (1) fewer acute stroke patients visiting the hospital freed up more of the medical workforce; (2) fewer acute stroke patients visiting the hospital led to a decrease in waiting times for patients to use medical devices; and (3) hospital policy during the COVID-19 period was tightened, requiring medical staff to be on high alert, taking fewer vacations and working more hours (23).

During the COVID-19 period, many acute stroke patients, especially with mild symptoms, were unwilling to seek treatment in hospitals, especially when the hospital also served as a designated COVID-19 center; this greatly delayed treatment and was not conducive to the prognosis of acute stroke patients. However, throughout our study, we found that such stroke centers tend to work more efficiently during the pandemic, and patients are more likely to have good prognoses. These designated hospitals have strict COVID-19 isolation wards, COVID-19 special channels, disinfection equipment, and inspection measures (24). Therefore, the possibility of nosocomial infection of COVID-19 is perhaps low. Hence, we recommend that acute stroke patients should visit the stroke center as soon as possible during the COVID-19 period. A hospital that also serves as a designated COVID-19 hospital will not be detrimental for acute stroke patients, but rather enable them to seek better medical services.

As of April 23, 2021, there were hundreds of millions of confirmed COVID-19 patients in the international community including the United States, Europe, India, and so on (1). What's worse, India was adding thousands of new confirmed cases of COVID-19 every day. These countries appear to be facing the same serious COVID-19 situation that China faced in its early days (25). Because of the large number of COVID-19 patients in these countries, there would be a huge demand of designated hospitals for centralized treatment of COVID-19 patients (26). Most of these hospitals also served as local acute stroke centers (27). Therefore, this study can provide an experience for designated COVID-19 hospitals in the international community when they are also responsible for the treatment of acute stroke.

Our study has some limitations. This was a single-center retrospective study, which may not be generalizable to other stroke centers. Additionally, the sample size was relatively less to reliably detect small but potentially important differences. In addition, our center admitted only COVID-19-negative patients, thus contributing to the decrease in stroke admission. Indeed, COVID-19 patients with stroke might have been admitted to other hospitals. Besides, only in-hospital times were considered in this study; however, the pandemic could also have had an impact on prehospital patient transportation that could have affected stroke admissions. Moreover, we used administrative coding data for the diagnosis of stroke, which was heavily influenced by the stroke neurologist's decision. However, through periodic follow-up data, this study greatly enhanced applications (28). Finally, only the current clinical status has been reported, and long-term clinical outcomes remain to be seen. The differences between designated and non-designated hospitals are also worth further study.

In conclusion, our study showed that fewer acute stroke patients, especially with mild symptoms, sought medical care in the designated hospitals during the COVID-19 period. Additionally, we found that this kind of designated COVID-19 hospital was more efficient for timely treatment of acute stroke during COVID-19. To avoid delayed treatment and obtain better treatment outcomes, we suggest that acute stroke patients should seek treatment at the nearest stroke center as soon as possible, even if the hospital also serves as a designated COVID-19 center.

## Data Availability Statement

The original contributions presented in the study are included in the article/supplementary material, further inquiries can be directed to the corresponding author/s.

## Ethics Statement

The studies involving human participants were reviewed and approved by the Medical Ethics Committee of Yongchuan Hospital Affiliated to Chongqing Medical University. The patients/participants provided their written informed consent to participate in this study. Written informed consent was obtained from the individual(s) for the publication of any potentially identifiable images or data included in this article.

## Author Contributions

QT: study design and data collection and analysis. Q-JL and L-BZ: study design. W-HF and X-YD: revise the paper. LW, H-MG, JW, RZ, and ML: data analysis. All authors contributed to the article and approved the submitted version.

## Funding

This work was supported by the Foundation and Frontier Program of Chongqing Science and Technology Commission (cstc2018jcyjAX0341), the Chongqing Key Program of Technological Innovation and Application Development (cstc2019jscx-gksbX0064); and the Medical Research Program of Chongqing Science and Technology Commission (2018ZDXM023).

## Conflict of Interest

The authors declare that the research was conducted in the absence of any commercial or financial relationships that could be construed as a potential conflict of interest.

## Publisher's Note

All claims expressed in this article are solely those of the authors and do not necessarily represent those of their affiliated organizations, or those of the publisher, the editors and the reviewers. Any product that may be evaluated in this article, or claim that may be made by its manufacturer, is not guaranteed or endorsed by the publisher.
